# 1-(5-Benzyl­sulfanyl-2,2-dimethyl-2,3-dihydro-1,3,4-thia­diazol-3-yl)-2,2-dimethyl­propan-1-one

**DOI:** 10.1107/S1600536812012639

**Published:** 2012-03-31

**Authors:** Mohd Sukeri Mohd Yusof, Fatimah Abdul Mutalib, Suhana Arshad, Ibrahim Abdul Razak

**Affiliations:** a Department of Chemical Sciences, Faculty of Science and Technology, Universiti Malaysia Terengganu, Mengabang Telipot, 21030 Kuala Terengganu, Malaysia; b School of Physics, Universiti Sains Malaysia, 11800 USM, Penang, Malaysia

## Abstract

In the title compound, C_16_H_22_N_2_OS_2_, the S atom of the thia­diazole ring and the attached methyl groups are disordered over two orientations with a refined site-occupancy ratio of 0.641 (11):0.359 (11). The thia­diazole ring is in a twist conformation in both disorder components. The mean plane through the thia­diazole ring makes dihedral angles of 77.39 (8) (major component) and 67.45 (11)° (minor component) with the benzene ring. Intra­molecular C—H⋯N inter­actions generate two *S*(6) ring motifs. In the crystal, mol­ecules are linked by C—H⋯O hydrogen bonds into zigzag chains parallel to the *b* axis.

## Related literature

For background to the pharmacological properties of thia­diazole derivatives, see: Noolvi *et al.* (2011 ▶); Yusuf *et al.* (2008 ▶). For a related structure, see: Fun *et al.* (2011 ▶). For hydrogen-bond motifs, see: Bernstein *et al.* (1995 ▶). For the stability of the temperature controller used in the data collection, see: Cosier & Glazer (1986 ▶). For ring conformations, see: Cremer & Pople (1975 ▶).
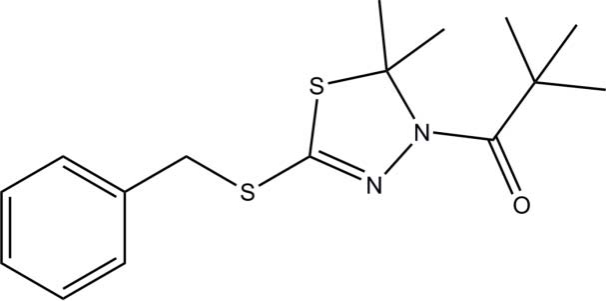


## Experimental

### 

#### Crystal data

C_16_H_22_N_2_OS_2_
*M* *_r_* = 322.48
Monoclinic, 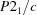

*a* = 16.6174 (2) Å
*b* = 10.5178 (1) Å
*c* = 9.6758 (1) Å
β = 96.345 (1)°
*V* = 1680.76 (3) Å^3^
*Z* = 4
Mo *K*α radiation
μ = 0.32 mm^−1^
*T* = 100 K
0.26 × 0.19 × 0.12 mm

#### Data collection

Bruker SMART APEXII CCD area-detector diffractometer
Absorption correction: multi-scan (*SADABS*; Bruker, 2009 ▶) *T*_min_ = 0.922, *T*_max_ = 0.962
22216 measured reflections
5972 independent reflections
4678 reflections with *I* > 2σ(*I*)
*R*_int_ = 0.033

#### Refinement

*R*[*F* ^2^ > 2σ(*F* ^2^)] = 0.039
*wR*(*F* ^2^) = 0.092
*S* = 1.02
5972 reflections
225 parameters
H-atom parameters constrained
Δρ_max_ = 0.38 e Å^−3^
Δρ_min_ = −0.25 e Å^−3^

### 

Data collection: *APEX2* (Bruker, 2009 ▶); cell refinement: *SAINT* (Bruker, 2009 ▶); data reduction: *SAINT*; program(s) used to solve structure: *SHELXTL* (Sheldrick, 2008 ▶); program(s) used to refine structure: *SHELXTL*; molecular graphics: *SHELXTL*; software used to prepare material for publication: *SHELXTL* and *PLATON* (Spek, 2009 ▶).

## Supplementary Material

Crystal structure: contains datablock(s) global, I. DOI: 10.1107/S1600536812012639/rz2718sup1.cif

Structure factors: contains datablock(s) I. DOI: 10.1107/S1600536812012639/rz2718Isup2.hkl

Supplementary material file. DOI: 10.1107/S1600536812012639/rz2718Isup3.cml

Additional supplementary materials:  crystallographic information; 3D view; checkCIF report

## Figures and Tables

**Table 1:** Hydrogen-bond geometry (Å, °)

*D*—H⋯*A*	*D*—H	H⋯*A*	*D*⋯*A*	*D*—H⋯*A*
C14—H14*B*⋯N1	0.98	2.36	2.9893 (15)	122
C15—H15*B*⋯N1	0.98	2.37	2.9803 (15)	120
C11—H11*B*⋯O1^i^	0.98	2.56	3.490 (4)	159

Symmetry code: (i) 
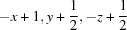
.

**Fig. 1.**
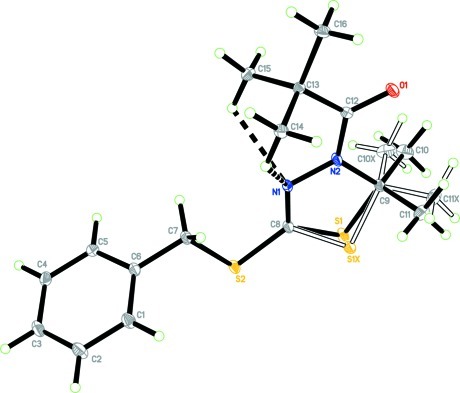
The molecular structure of the title compound with 30% probability displacement ellipsoids. Dashed lines indicate intramolecular hydrogen bonds. Bonds involving the minor component of the disorder are shown as empty sticks.

**Fig. 2.**
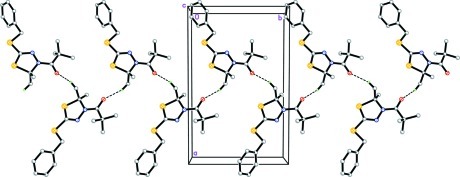
The crystal packing of the title compound viewed along the c axis. The H atoms not involved in the intermolecular interactions (dashed lines) are omitted for clarity. Only major disordered components are shown.

## supplementary crystallographic information

### Comment

Thiadiazole derivatives have been reported to posses anti-cancer (Noolvi *et al.*, 2011) and anti-depressant activity (Yusuf *et al.*, 2008). The title compound is one of these thiadiazole derivatives, and its crystal structure is reported herein.

In the molecule of the title compound (Fig. 1), the S atom of the thiadiazole ring and the attached dimethyl groups (C10/C10X and C11/C11X) are disordered over two orientations with a refined site-occupancy ratio of 0.641 (11):0.359 (11). The disordered thiadiazole (S1/N1/N2/C8/C9 and S1X/N1/N2/C8/C9) rings are both in twist conformation (Cremer & Pople, 1975) in which the ring is twisted about the C9–S1 bond [puckering parameters: *Q* = 0.1477 (19) Å and φ= 167.7 (5)°] and about the S1X–C8 bond [puckering parameters: *Q* = 0.131 (2) Å and φ= 298.6 (8)°], respectively. The mean plane through the thiadiazole rings make dihedral angles of 77.39 (8) and 67.45 (11)°, respectively, with the benzene (C1–C6) ring. Intramolecular C14—H14B···N1 and C15—H15B···N1 interactions (Table 1) generate two *S*(6) ring motifs (Bernstein *et al.*, 1995). The bond lengths and angles are within normal ranges and are comparable to those reported in a related structure (Fun *et al.*, 2011). The crystal packing is shown in Fig. 2. Intermolecular C11—H11B···O1 (Table 1) hydrogen bonds link the molecules into one dimensional zigzag chains parallel to the *b* axis.

### Experimental

A solution of pivaloylisothiocyanate (1.0 g, 8 mmol) in 30 ml acetone was added into a flask containing 30 ml acetone solution of s-benzyldithiocarbazate (1.5 g, 8.00 mmol). The mixture was refluxed for 4 h, then, the solution was filtered-off and left to evaporate at room temperature. Colourless crystals suitable for X-ray analyisis were obtained after one day on slow evaporation of the solvent (yield 60%, *M.p.* 503.5–504.5 K, IR(KBr)cm^-1^: 1334.72 (ν_C—N_), 1547.95 (ν_C__═__N_), 1647.08 (ν_C__═__O_), 8944.79 (ν_C—S_). ^1^H NMR (CDCl_3_)δp.p.m. 1.289 (s, 9H, -(CH_3_)_3_), 2.004 (s, 6H, -(CH_3_)_2_), 4.330 (s, 2H, –CH_2_), 7.35–7.45 (m, 2H, ar-H). ^13^C NMR (CDCl_3_)δp.p.m. 127.86–135.40 (6 C, ar-C), 144.57 (thiadiazole carbon), 176.72 (C═O), 27.06–37.50(4 C, –C-(CH_3_)_3_). Anal. Found (calc.) for C_16_H_22_N_2_OS_2_ (%): C, 59.59(58.98); H, 6.88(6.86); N, 8.69(8.66); S, 19.89(19.86).

### Refinement

The S atom of the thiadiazole ring and the attached dimethyl groups (C10/C10X) and C11/C11X) are disordered over two orientations with a refined site-occupancy ratio of 0.641 (11):0.359 (11). All H atoms were positioned geometrically [C–H = 0.95–0.99 Å] and refined using a riding model with *U*_iso_(H) = 1.2 or 1.5 *U*_eq_(C). A rotating group model was applied to the methyl groups.

### Crystal data

**Table:**

C_16_H_22_N_2_OS_2_	*F*(000) = 688
*M**_r_* = 322.48	*D*_x_ = 1.274 Mg m^−^^3^
Monoclinic, *P*2_1_/*c*	Mo *K*α radiation, λ = 0.71073 Å
Hall symbol: -P 2ybc	Cell parameters from 6904 reflections
*a* = 16.6174 (2) Å	θ = 2.9–32.3°
*b* = 10.5178 (1) Å	µ = 0.32 mm^−^^1^
*c* = 9.6758 (1) Å	*T* = 100 K
β = 96.345 (1)°	Block, colourless
*V* = 1680.76 (3) Å^3^	0.26 × 0.19 × 0.12 mm
*Z* = 4	

### Data collection

**Table:**

Bruker SMART APEXII CCD area-detector diffractometer	5972 independent reflections
Radiation source: fine-focus sealed tube	4678 reflections with *I* > 2σ(*I*)
Graphite monochromator	*R*_int_ = 0.033
φ and ω scans	θ_max_ = 32.4°, θ_min_ = 2.3°
Absorption correction: multi-scan (*SADABS*; Bruker, 2009)	*h* = −25→13
*T*_min_ = 0.922, *T*_max_ = 0.962	*k* = −15→15
22216 measured reflections	*l* = −12→14

### Refinement

**Table:**

Refinement on *F*^2^	Primary atom site location: structure-invariant direct methods
Least-squares matrix: full	Secondary atom site location: difference Fourier map
*R*[*F*^2^ > 2σ(*F*^2^)] = 0.039	Hydrogen site location: inferred from neighbouring sites
*wR*(*F*^2^) = 0.092	H-atom parameters constrained
*S* = 1.02	*w* = 1/[σ^2^(*F*_o_^2^) + (0.0355*P*)^2^ + 0.5012*P*] where *P* = (*F*_o_^2^ + 2*F*_c_^2^)/3
5972 reflections	(Δ/σ)_max_ = 0.001
225 parameters	Δρ_max_ = 0.38 e Å^−^^3^
0 restraints	Δρ_min_ = −0.25 e Å^−^^3^

### Special details

**Table:**

Experimental. The crystal was placed in the cold stream of an Oxford Cryosystems Cobra open-flow nitrogen cryostat (Cosier & Glazer, 1986) operating at 100.0 (1) K.
Geometry. All e.s.d.'s (except the e.s.d. in the dihedral angle between two l.s. planes) are estimated using the full covariance matrix. The cell e.s.d.'s are taken into account individually in the estimation of e.s.d.'s in distances, angles and torsion angles; correlations between e.s.d.'s in cell parameters are only used when they are defined by crystal symmetry. An approximate (isotropic) treatment of cell e.s.d.'s is used for estimating e.s.d.'s involving l.s. planes.
Refinement. Refinement of *F*^2^ against ALL reflections. The weighted *R*-factor *wR* and goodness of fit *S* are based on *F*^2^, conventional *R*-factors *R* are based on *F*, with *F* set to zero for negative *F*^2^. The threshold expression of *F*^2^ > σ(*F*^2^) is used only for calculating *R*-factors(gt) *etc*. and is not relevant to the choice of reflections for refinement. *R*-factors based on *F*^2^ are statistically about twice as large as those based on *F*, and *R*- factors based on ALL data will be even larger.

### Fractional atomic coordinates and isotropic or equivalent isotropic displacement parameters (Å^2^)

**Table:**

	*x*	*y*	*z*	*U*_iso_*/*U*_eq_	Occ. (<1)
S1	0.36774 (15)	0.26997 (19)	0.2500 (3)	0.0233 (4)	0.641 (11)
S1X	0.3827 (2)	0.28188 (18)	0.2089 (6)	0.0216 (6)	0.359 (11)
S2	0.214397 (18)	0.34659 (3)	0.07503 (3)	0.01843 (7)	
O1	0.41502 (5)	−0.14077 (8)	0.14798 (9)	0.02125 (18)	
N1	0.28175 (6)	0.11644 (9)	0.08318 (10)	0.01500 (18)	
N2	0.34822 (6)	0.04481 (9)	0.13754 (10)	0.01761 (19)	
C1	0.09412 (8)	0.41815 (12)	−0.21819 (12)	0.0227 (2)	
H1A	0.1467	0.4243	−0.2475	0.027*	
C2	0.03192 (8)	0.49319 (13)	−0.28083 (14)	0.0279 (3)	
H2A	0.0420	0.5505	−0.3528	0.033*	
C3	−0.04488 (8)	0.48490 (12)	−0.23872 (14)	0.0250 (3)	
H3A	−0.0875	0.5360	−0.2822	0.030*	
C4	−0.05940 (7)	0.40174 (11)	−0.13292 (13)	0.0223 (2)	
H4A	−0.1120	0.3961	−0.1036	0.027*	
C5	0.00308 (7)	0.32658 (11)	−0.06983 (12)	0.0193 (2)	
H5A	−0.0070	0.2701	0.0029	0.023*	
C6	0.08002 (7)	0.33358 (10)	−0.11244 (11)	0.0166 (2)	
C7	0.14763 (7)	0.25122 (11)	−0.04636 (12)	0.0183 (2)	
H7A	0.1786	0.2155	−0.1190	0.022*	
H7B	0.1250	0.1798	0.0036	0.022*	
C8	0.28772 (7)	0.23196 (10)	0.12453 (11)	0.0161 (2)	
C9	0.41461 (7)	0.11385 (10)	0.22346 (11)	0.0159 (2)	
C10	0.4419 (3)	0.0577 (4)	0.3624 (4)	0.0267 (7)	0.641 (11)
H10A	0.4695	−0.0232	0.3500	0.040*	0.641 (11)
H10B	0.3949	0.0431	0.4131	0.040*	0.641 (11)
H10C	0.4794	0.1165	0.4153	0.040*	0.641 (11)
C10X	0.4118 (6)	0.0686 (8)	0.3771 (8)	0.0296 (14)	0.359 (11)
H10D	0.4294	−0.0202	0.3860	0.044*	0.359 (11)
H10E	0.3563	0.0760	0.4016	0.044*	0.359 (11)
H10F	0.4479	0.1218	0.4397	0.044*	0.359 (11)
C11	0.4847 (2)	0.1317 (4)	0.1339 (4)	0.0252 (7)	0.641 (11)
H11A	0.5099	0.0491	0.1195	0.038*	0.641 (11)
H11B	0.5251	0.1892	0.1814	0.038*	0.641 (11)
H11C	0.4639	0.1680	0.0438	0.038*	0.641 (11)
C11X	0.4988 (4)	0.1030 (7)	0.1826 (10)	0.0275 (14)	0.359 (11)
H11D	0.5184	0.0158	0.1986	0.041*	0.359 (11)
H11E	0.5346	0.1620	0.2385	0.041*	0.359 (11)
H11F	0.4983	0.1242	0.0839	0.041*	0.359 (11)
C12	0.35519 (7)	−0.08096 (10)	0.10018 (11)	0.0146 (2)	
C13	0.28826 (7)	−0.14282 (10)	−0.00111 (11)	0.0147 (2)	
C14	0.28175 (7)	−0.07640 (11)	−0.14377 (11)	0.0178 (2)	
H14A	0.3346	−0.0783	−0.1799	0.027*	
H14B	0.2648	0.0120	−0.1336	0.027*	
H14C	0.2417	−0.1207	−0.2086	0.027*	
C15	0.20567 (7)	−0.14025 (11)	0.05629 (12)	0.0202 (2)	
H15A	0.1667	−0.1908	−0.0041	0.030*	
H15B	0.1865	−0.0523	0.0592	0.030*	
H15C	0.2112	−0.1760	0.1504	0.030*	
C16	0.31270 (8)	−0.28173 (10)	−0.02075 (13)	0.0210 (2)	
H16A	0.3649	−0.2846	−0.0588	0.031*	
H16B	0.2715	−0.3239	−0.0852	0.031*	
H16C	0.3173	−0.3255	0.0692	0.031*	

### Atomic displacement parameters (Å^2^)

**Table:**

	*U*^11^	*U*^22^	*U*^33^	*U*^12^	*U*^13^	*U*^23^
S1	0.0205 (5)	0.0179 (4)	0.0287 (7)	0.0056 (3)	−0.0097 (5)	−0.0087 (4)
S1X	0.0196 (8)	0.0125 (4)	0.0302 (13)	−0.0001 (4)	−0.0078 (8)	−0.0024 (6)
S2	0.01715 (14)	0.01447 (12)	0.02242 (14)	0.00448 (10)	−0.00341 (11)	−0.00322 (10)
O1	0.0199 (4)	0.0168 (4)	0.0253 (4)	0.0047 (3)	−0.0053 (3)	−0.0004 (3)
N1	0.0120 (4)	0.0150 (4)	0.0175 (4)	0.0024 (3)	−0.0007 (3)	0.0005 (3)
N2	0.0152 (5)	0.0142 (4)	0.0215 (5)	0.0033 (3)	−0.0065 (4)	−0.0029 (3)
C1	0.0196 (6)	0.0273 (6)	0.0219 (6)	0.0068 (5)	0.0048 (5)	0.0048 (5)
C2	0.0276 (7)	0.0321 (7)	0.0243 (6)	0.0092 (5)	0.0042 (5)	0.0108 (5)
C3	0.0204 (6)	0.0257 (6)	0.0277 (6)	0.0078 (5)	−0.0030 (5)	0.0033 (5)
C4	0.0147 (5)	0.0203 (5)	0.0314 (6)	0.0012 (4)	0.0002 (5)	−0.0008 (5)
C5	0.0177 (6)	0.0171 (5)	0.0227 (5)	−0.0007 (4)	0.0003 (4)	0.0007 (4)
C6	0.0167 (5)	0.0152 (5)	0.0168 (5)	0.0029 (4)	−0.0024 (4)	−0.0018 (4)
C7	0.0169 (5)	0.0163 (5)	0.0204 (5)	0.0027 (4)	−0.0037 (4)	−0.0021 (4)
C8	0.0149 (5)	0.0157 (5)	0.0171 (5)	0.0023 (4)	−0.0013 (4)	−0.0013 (4)
C9	0.0143 (5)	0.0144 (4)	0.0180 (5)	0.0018 (4)	−0.0029 (4)	−0.0025 (4)
C10	0.037 (2)	0.0245 (11)	0.0163 (13)	−0.0004 (14)	−0.0060 (14)	−0.0003 (9)
C10X	0.043 (4)	0.025 (2)	0.020 (2)	−0.009 (3)	0.001 (3)	0.0002 (17)
C11	0.0236 (14)	0.0246 (14)	0.0280 (16)	−0.0058 (10)	0.0061 (12)	−0.0046 (11)
C11X	0.020 (2)	0.024 (2)	0.040 (4)	−0.0046 (18)	0.007 (2)	−0.010 (2)
C12	0.0162 (5)	0.0134 (4)	0.0142 (5)	0.0011 (4)	0.0018 (4)	0.0008 (4)
C13	0.0153 (5)	0.0136 (4)	0.0149 (5)	−0.0005 (4)	0.0007 (4)	−0.0004 (4)
C14	0.0199 (6)	0.0182 (5)	0.0147 (5)	−0.0004 (4)	−0.0004 (4)	−0.0001 (4)
C15	0.0178 (6)	0.0202 (5)	0.0229 (5)	−0.0037 (4)	0.0043 (5)	−0.0005 (4)
C16	0.0258 (6)	0.0142 (5)	0.0223 (5)	0.0004 (4)	−0.0004 (5)	−0.0015 (4)

### Geometric parameters (Å, º)

**Table:**

S1—C8	1.7448 (16)	C9—C11	1.539 (3)
S1—C9	1.8473 (15)	C9—C10X	1.566 (8)
S1X—C8	1.774 (2)	C10—H10A	0.9800
S1X—C9	1.846 (2)	C10—H10B	0.9800
S2—C8	1.7432 (11)	C10—H10C	0.9800
S2—C7	1.8245 (12)	C10X—H10D	0.9800
O1—C12	1.2233 (13)	C10X—H10E	0.9800
N1—C8	1.2795 (14)	C10X—H10F	0.9800
N1—N2	1.3913 (13)	C11—H11A	0.9800
N2—C12	1.3796 (14)	C11—H11B	0.9800
N2—C9	1.4942 (14)	C11—H11C	0.9800
C1—C2	1.3857 (17)	C11X—H11D	0.9800
C1—C6	1.3950 (16)	C11X—H11E	0.9800
C1—H1A	0.9500	C11X—H11F	0.9800
C2—C3	1.3847 (18)	C12—C13	1.5428 (15)
C2—H2A	0.9500	C13—C16	1.5339 (15)
C3—C4	1.3878 (18)	C13—C15	1.5365 (16)
C3—H3A	0.9500	C13—C14	1.5401 (15)
C4—C5	1.3912 (17)	C14—H14A	0.9800
C4—H4A	0.9500	C14—H14B	0.9800
C5—C6	1.3880 (16)	C14—H14C	0.9800
C5—H5A	0.9500	C15—H15A	0.9800
C6—C7	1.5042 (16)	C15—H15B	0.9800
C7—H7A	0.9900	C15—H15C	0.9800
C7—H7B	0.9900	C16—H16A	0.9800
C9—C10	1.492 (4)	C16—H16B	0.9800
C9—C11X	1.499 (6)	C16—H16C	0.9800
			
C8—S1—C9	90.02 (7)	C11X—C9—S1	121.6 (2)
C8—S1X—C9	89.16 (10)	C11—C9—S1	109.09 (13)
C8—S2—C7	98.83 (5)	C10X—C9—S1	94.7 (3)
C8—N1—N2	111.41 (9)	C9—C10—H10A	109.5
C12—N2—N1	120.42 (9)	C9—C10—H10B	109.5
C12—N2—C9	122.25 (9)	C9—C10—H10C	109.5
N1—N2—C9	116.98 (8)	C9—C10X—H10D	109.5
C2—C1—C6	120.42 (12)	C9—C10X—H10E	109.5
C2—C1—H1A	119.8	H10D—C10X—H10E	109.5
C6—C1—H1A	119.8	C9—C10X—H10F	109.5
C3—C2—C1	120.18 (12)	H10D—C10X—H10F	109.5
C3—C2—H2A	119.9	H10E—C10X—H10F	109.5
C1—C2—H2A	119.9	C9—C11—H11A	109.5
C2—C3—C4	119.85 (11)	C9—C11—H11B	109.5
C2—C3—H3A	120.1	C9—C11—H11C	109.5
C4—C3—H3A	120.1	C9—C11X—H11D	109.5
C3—C4—C5	119.98 (12)	C9—C11X—H11E	109.5
C3—C4—H4A	120.0	H11D—C11X—H11E	109.5
C5—C4—H4A	120.0	C9—C11X—H11F	109.5
C6—C5—C4	120.45 (11)	H11D—C11X—H11F	109.5
C6—C5—H5A	119.8	H11E—C11X—H11F	109.5
C4—C5—H5A	119.8	O1—C12—N2	118.84 (10)
C5—C6—C1	119.12 (11)	O1—C12—C13	121.47 (9)
C5—C6—C7	120.83 (10)	N2—C12—C13	119.69 (9)
C1—C6—C7	120.06 (11)	C16—C13—C15	108.72 (9)
C6—C7—S2	109.24 (8)	C16—C13—C14	108.32 (9)
C6—C7—H7A	109.8	C15—C13—C14	109.81 (9)
S2—C7—H7A	109.8	C16—C13—C12	107.35 (9)
C6—C7—H7B	109.8	C15—C13—C12	111.90 (9)
S2—C7—H7B	109.8	C14—C13—C12	110.64 (9)
H7A—C7—H7B	108.3	C13—C14—H14A	109.5
N1—C8—S2	122.90 (9)	C13—C14—H14B	109.5
N1—C8—S1	117.51 (10)	H14A—C14—H14B	109.5
S2—C8—S1	119.24 (7)	C13—C14—H14C	109.5
N1—C8—S1X	117.33 (12)	H14A—C14—H14C	109.5
S2—C8—S1X	118.74 (9)	H14B—C14—H14C	109.5
C10—C9—N2	116.2 (2)	C13—C15—H15A	109.5
C10—C9—C11X	90.4 (3)	C13—C15—H15B	109.5
N2—C9—C11X	118.0 (3)	H15A—C15—H15B	109.5
C10—C9—C11	112.43 (18)	C13—C15—H15C	109.5
N2—C9—C11	107.73 (16)	H15A—C15—H15C	109.5
N2—C9—C10X	106.4 (3)	H15B—C15—H15C	109.5
C11X—C9—C10X	110.7 (3)	C13—C16—H16A	109.5
N2—C9—S1X	103.56 (9)	C13—C16—H16B	109.5
C11X—C9—S1X	108.7 (2)	H16A—C16—H16B	109.5
C10X—C9—S1X	109.0 (3)	C13—C16—H16C	109.5
C10—C9—S1	108.51 (15)	H16A—C16—H16C	109.5
N2—C9—S1	102.27 (8)	H16B—C16—H16C	109.5
			
C8—N1—N2—C12	−176.95 (10)	N1—N2—C9—C11	−103.8 (2)
C8—N1—N2—C9	−3.69 (13)	C12—N2—C9—C10X	−77.1 (4)
C6—C1—C2—C3	0.1 (2)	N1—N2—C9—C10X	109.8 (4)
C1—C2—C3—C4	0.4 (2)	C12—N2—C9—S1X	168.1 (2)
C2—C3—C4—C5	−0.26 (19)	N1—N2—C9—S1X	−5.0 (3)
C3—C4—C5—C6	−0.34 (18)	C12—N2—C9—S1	−175.75 (17)
C4—C5—C6—C1	0.79 (17)	N1—N2—C9—S1	11.12 (18)
C4—C5—C6—C7	−179.04 (11)	C8—S1X—C9—C10	−123.1 (4)
C2—C1—C6—C5	−0.66 (18)	C8—S1X—C9—N2	8.7 (3)
C2—C1—C6—C7	179.17 (12)	C8—S1X—C9—C11X	135.0 (5)
C5—C6—C7—S2	−103.10 (11)	C8—S1X—C9—C11	118.0 (4)
C1—C6—C7—S2	77.07 (12)	C8—S1X—C9—C10X	−104.3 (5)
C8—S2—C7—C6	−176.36 (8)	C8—S1X—C9—S1	−78.5 (3)
N2—N1—C8—S2	179.94 (8)	C8—S1—C9—C10	−134.8 (3)
N2—N1—C8—S1	−7.0 (2)	C8—S1—C9—N2	−11.51 (19)
N2—N1—C8—S1X	11.7 (3)	C8—S1—C9—C11X	122.7 (5)
C7—S2—C8—N1	−4.34 (11)	C8—S1—C9—C11	102.4 (3)
C7—S2—C8—S1	−177.32 (18)	C8—S1—C9—C10X	−119.5 (4)
C7—S2—C8—S1X	163.8 (3)	C8—S1—C9—S1X	84.9 (3)
C9—S1—C8—N1	11.8 (2)	N1—N2—C12—O1	178.79 (10)
C9—S1—C8—S2	−174.86 (9)	C9—N2—C12—O1	5.89 (16)
C9—S1—C8—S1X	−81.9 (3)	N1—N2—C12—C13	−0.76 (15)
C9—S1X—C8—N1	−12.7 (3)	C9—N2—C12—C13	−173.67 (9)
C9—S1X—C8—S2	178.57 (12)	O1—C12—C13—C16	1.05 (14)
C9—S1X—C8—S1	82.3 (3)	N2—C12—C13—C16	−179.41 (10)
C12—N2—C9—C10	−57.8 (3)	O1—C12—C13—C15	120.25 (11)
N1—N2—C9—C10	129.1 (2)	N2—C12—C13—C15	−60.21 (13)
C12—N2—C9—C11X	48.0 (5)	O1—C12—C13—C14	−116.95 (11)
N1—N2—C9—C11X	−125.2 (4)	N2—C12—C13—C14	62.59 (13)
C12—N2—C9—C11	69.4 (2)		

### Hydrogen-bond geometry (Å, º)

**Table:**

*D*—H···*A*	*D*—H	H···*A*	*D*···*A*	*D*—H···*A*
C14—H14*B*···N1	0.98	2.36	2.9893 (15)	122
C15—H15*B*···N1	0.98	2.37	2.9803 (15)	120
C11—H11*B*···O1^i^	0.98	2.56	3.490 (4)	159

Symmetry code: (i) −*x*+1, *y*+1/2, −*z*+1/2.
